# Lipidomic Profiling of the Epidermis in a Mouse Model of Dermatitis Reveals Sexual Dimorphism and Changes in Lipid Composition before the Onset of Clinical Disease

**DOI:** 10.3390/metabo10070299

**Published:** 2020-07-21

**Authors:** Jackeline Franco, Bartek Rajwa, Christina R. Ferreira, John P. Sundberg, Harm HogenEsch

**Affiliations:** 1 Department of Comparative Pathobiology, Purdue University, West Lafayette, IN 47907, USA; francoj@purdue.edu; 2 Bindley Bioscience Center, Purdue University, West Lafayette, IN 47907, USA; 3 Metabolite Profiling Facility, Bindley Bioscience Center, Purdue University, West Lafayette, IN 47907, USA; cferrei@purdue.edu; 4 The Jackson Laboratory, Bar Harbor, ME 04609, USA; john.sundberg@jax.org; 5 Purdue Institute of Inflammation, Immunology and Infectious Diseases, Purdue University, West Lafayette, IN 47907, USA

**Keywords:** lipidomics, atopic dermatitis, SHARPIN-deficient mice, flow-injection mass-spectrometry, predictive elastic net

## Abstract

Atopic dermatitis (AD) is a multifactorial disease associated with alterations in lipid composition and organization in the epidermis. Multiple variants of AD exist with different outcomes in response to therapies. The evaluation of disease progression and response to treatment are observational assessments with poor inter-observer agreement highlighting the need for molecular markers. SHARPIN-deficient mice (*Sharpin^cpdm^*) spontaneously develop chronic proliferative dermatitis with features similar to AD in humans. To study the changes in the epidermal lipid-content during disease progression, we tested 72 epidermis samples from three groups (5-, 7-, and 10-weeks old) of *cpdm* mice and their WT littermates. An agnostic mass-spectrometry strategy for biomarker discovery termed multiple-reaction monitoring (MRM)-profiling was used to detect and monitor 1,030 lipid ions present in the epidermis samples. In order to select the most relevant ions, we utilized a two-tiered filter/wrapper feature-selection strategy. Lipid categories were compressed, and an elastic-net classifier was used to rank and identify the most predictive lipid categories for sex, phenotype, and disease stages of *cpdm* mice. The model accurately classified the samples based on phospholipids, cholesteryl esters, acylcarnitines, and sphingolipids, demonstrating that disease progression cannot be defined by one single lipid or lipid category.

## 1. Introduction

Atopic dermatitis (AD) is a multifactorial inflammatory skin disease that affects people and domestic animals worldwide [1]. Multiple variants (endotypes) of AD occur based on differences in the genetic background of patients, environment, immune activation pathways, and epidermal barrier status [1,2,3]. The classical AD presentation includes increased IgE serum levels, increased concentration of type 2 cytokines [4,5], and filaggrin (*FLG*) mutations that underlie skin barrier dysfunction [6,7,8]. However, variants of AD with normal levels of serum IgE and an increase of Th22 and Th17 cytokines instead of type 2 cytokines also exist [7,9]. In addition, *FLG* mutations occur in only 10% to 30% of AD patients [10,11]. The less common variants of AD may require different therapeutic approaches as standard forms of therapy could result in unsatisfactory outcomes. Currently, clinical assessment of disease severity and diagnosis of AD relies on subjective observation of clinical signs, which change with the chronicity of the disease phase [6,7]. Several assessment indices are used to diagnose and score the disease, but these have poor inter-observer agreement highlighting the need for molecular disease biomarkers [12,13,14,15].

Alterations in the skin lipid composition have been reported in AD patients regardless of the genetic background, immune response, and clinical presentation [16,17]. Investigation of the lipid composition of the stratum corneum of the skin across different analytical platforms revealed changes in ceramide (CER) structure and presence of shorter and more unsaturated free fatty acids (FFA) in AD patients compared to healthy subjects [18,19,20,21]. Others reported changes in the amounts of phospholipids (PL), cholesteryl esters (CE), and triacylglycerides (TAG) in atopic skin, sweat, and sebum compared with healthy controls [22,23,24]. Alterations in the lipid composition lead to a disorganized stratum corneum lipid matrix and impaired barrier function of the skin [18], which permits increased allergen penetration that induces or aggravates the inflammatory reaction [25,26]. The cause of these lipid changes is not well understood, and it remains uncertain whether they result from a primary defect or downregulation of lipid processing enzymes by type 2 cytokines released in the course of dermatitis [27,28].

*Sharpin^cpdm^* mice (hereafter referred to as *cpdm* mice), which have a mutation that causes absence of the SHARPIN protein, develop a chronic proliferative dermatitis that is very similar to human AD. The condition is characterized by pruritus, alopecia, and thickening of the skin, as well as accumulation of eosinophils, mast cells, M2 macrophages, and increased expression of type 2 cytokines [29,30]. In a previous study, we identified specific changes in ceramides and fatty acids in the epidermis of female SHARPIN-deficient mice with chronic proliferative dermatitis using a novel accelerated mass spectrometry strategy, multiple reaction monitoring (MRM)-profiling [31]. As the severity of the dermatitis rapidly increases with age, *cpdm* mice present a suitable model to identify lipid changes in the skin before the onset of clinical signs of inflammation and during progression of the dermatitis.

Lipidomics allows the detection and identification of a large number of molecules in a high-throughput manner aimed at the identification of new biomarkers for diagnosis and disease progression as well as novel targets for treatment [32]. These systems biology approaches yield complicated, high-dimensional data that should not be analyzed using naive univariate statistical methods as they may produce a high false-positive rate when predicting and classifying phenotypes. Consequently, this data requires multivariate approaches [33,34].

Although predicting phenotype from lipidomic data can be performed using various machine learning approaches, the critical question asked by biologists searching for a mechanistic model is the meaning of the statistical prediction. The black box predictors may be entirely accurate, but they do not allow easy formation of post-classification hypotheses regarding the causal relationship between the employed features, and the produced prediction. On the other hand, ante-hoc explainable models such as regression-based approaches can be used not only for supervised classification but also for the identification of critically important covariates, which can be further studied in pursuit of a mechanistic model [35]. Therefore, feature selection and reduction employing methods such as elastic-net (ENET) regularized regression are beneficial for finding key predictive features in the rich biological data and for identifying potential biomarkers amid the vast number of responses produced by systems biology methodologies [36,37,38,39]. Here we report the postulated biomarkers of AD, delivered via a multi-tiered feature selection strategy that processed the data generated by MRM-profiling in order to characterize lipid changes in the skin before the onset of clinical signs, both at the level of lipid categories and individual lipids ions. The method was used to investigate the association of the identified features with disease progression in male and female *cpdm* mice and their age and sex-matched wild type (WT) littermates. The study identified alterations in lipid composition preceding the onset of clinical dermatitis and a subset of lipid ions predictive of the disease stage of each sample. Additionally, the data demonstrated that the epidermis of female and male mice had distinct lipid profiles and differed in the lipid changes associated with disease progression.

## 2. Results

### 2.1. Association of Sex and Genotype to the Lipid Composition of the Mice Skin

Epidermal samples (*n* = 72, 36 *cpdm,* and 36 WT) were monitored for the presence of 1030 lipid ions belonging to multiple lipid categories. First, the collected data were pre-processed as described in the Methods section by executing log-ratio transformations, followed by single decomposition value (SVD)-driven principal component analysis. The result was visualized in the compositional principal component (CPC) space.

**Figure 1:**
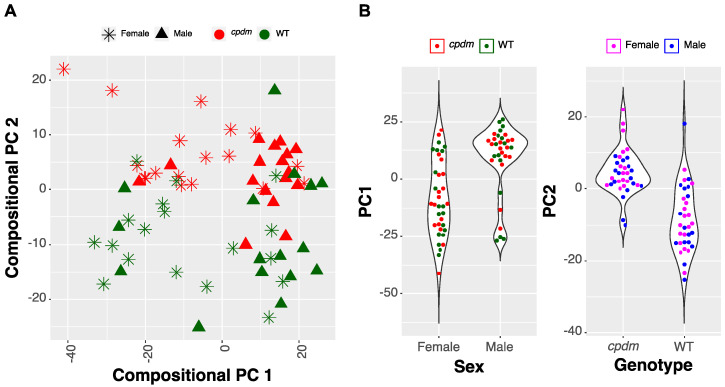
Monitored lipid ions in male and female *cpdm* and wild type (WT) epidermis by multiple reaction monitoring (MRM) scans in positive ion mode. Discrimination of the sex, as well as the genotypes of WT and *cpdm* mice (including non-lesional samples), was observed by compositional principal components (CPC) projection. (**A**) score plot of CPC analysis. (**B**) violin plots representing the separation of samples by sex and genotype. CPC 1 explained 30.2% of the variability of the data separating the samples by sex. CPC 2 explained 22.4% of the variance and was aligned with the genotype.

The CPC projection clearly differentiated samples by sex with the first component explaining 30.2% of the data variance, whereas the second component accounting for 22.4% of the variance was mostly associated with the genotype (Figure 1). The list of transitions driving the separation of samples in the CPC score plot is provided in Table S1.

The visualization demonstrates that the information regarding the sex and genotype is encoded in the lipidomic profile of the sample. However, the CPC projection does not provide an actionable input from the perspective of feature selection or causal explanation. The 296 lipids in the top 25-percentile of accounted variance in CPC 1 contribute only between 0.167 and 0.31 percent to the representation. Similarly, for CPC 2, the individual contribution of each lipid in the top 25-percentile group ranges from 0.137 to 0.37. Therefore, a feature selection strategy is necessary.

The feature selection involved a two-tier selection, including a univariate step followed by the creation of a feature-ranking ENET regression model able to separate the samples into classes based on sex and genotype. The analysis was performed assuming a binary case (for sex and genotype data). These tasks were approached using two different methods: Analysis of CPC-compressed compositional features representing lipid categories, and analysis of individual ions regardless of the category.

#### 2.1.1. Selection of Predictive Lipid Categories for Sex and Genotype

**Figure 2:**
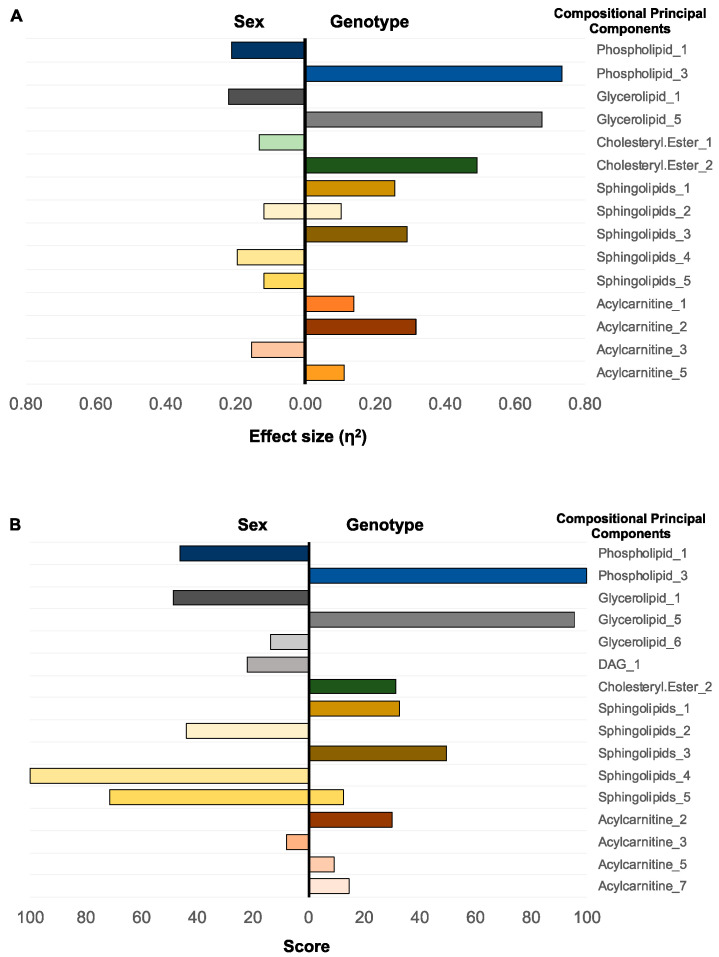
Importance of compressed categories for sex and genotype classification. (**A**) univariate linear model—selected lipid categories compositional principal component (CPC) based on their effect size (η^2^). (**B**) top 10 CPC of lipid categories ranked by the multivariate elastic net model for sex and genotype, which generate a prediction accuracy of 76% for the sex and 100% for the genotype.

The compressed features identified glycerolipids CPC 1, phospholipids CPC 1, and sphingolipids CPC 4 as most capable of separating samples by sex in the first selection step. For these features, the effect size expressed as η^2^ ranged from 0.12 to 0.22 (Figure 2A). The η^2^ of 0.22 is equivalent to Cohen’s *f* = 0.53, which in a univariate model with two groups is equal to Cohen’s *d* = 1.06, signifying a very substantial effect size. The subsequent feature-ranking ENET selected the sphingolipids CPC 4 and 5, phospholipids CPC 1, and glycerolipids CPC 1 as the most critical features to classify the samples by sex (Figure 2B). The classifier built using the 20-top composite CPC features had an overall accuracy of 0.76, CI_0.95_ = (0.74, 0.77).

The univariate selection of compressed features for the binary genotype classification (WT vs. *cpdm*) identified phospholipids CPC 3, glycerolipids CPC 5, cholesteryl esters CPC 2, acylcarnitine CPC 2, and sphingolipids CPC 3 and 1, as the most predictive. The observed η^2^ ranged from 0.26 to 0.73 for the top features (Figure 2A). The subsequently trained ENET identified phospholipids CPC 3, glycerolipids CPC 5, sphingolipids CPC 3 and 1, and cholesteryl esters CPC 2 as the top features in terms of importance (Figure 2B) and the model approached 100% accuracy (CI_0.95_ from 1 to 0.95).

These results demonstrate that the composition and abundance of the same lipid categories carry information regarding the sex and genotype status of the tested animals. Similar to the CPC visualization incorporating all the lipid ions simultaneously, we observed that the information regarding sex and genotype is present in multiple compositional principal components.

#### 2.1.2. Individual Lipid Ions Feature Selection for Genotype

Following the analysis of lipid categories, we performed a feature importance analysis for individual lipid ions. The study was conducted for genotypes (*cpdm* vs. WT) in a binary setting, filtering out the influence of sex. It is important to emphasize that even though this model used the individual lipid ion features, the tentative chemical attribution of the measured ions was not independently confirmed to eliminate the likelihood of isotopic interferences.

In the univariate step, we applied a linear model-based filter retaining only the features associated with genotype class (adjusted *p* < 0.01) and not strongly associated with sex (adjusted *p* > 0.05). The 100 lipid ions with the highest partial η^2^ (ranging from 0.26 to 0.76) were selected for further analysis. In the top-100 group, acylcarnitines and phospholipids were by far the most prevalent. However, among the top ten features, there were nine phospholipids and one ion associated with sphingolipids. Table 1 shows the highest-scoring lipid ions as well as their weak effect size associated with sex.

As in the previous analysis task, the second filtering step included an ENET regression used to filter and rank the lipids pre-selected by the univariate step. The trained ENET achieved an overall accuracy of 0.99, CI_95%_ = (0.924, 1). The most predictive lipid ions are summarized in Table 2. Among the selected lipids, five were phospholipids, two glycerolipids, and one was identified as a sphingolipid.

### 2.2. Selection of Features Associated with Disease Progression

#### 2.2.1. Compositional Principal Component Analysis and Data Visualization

**Figure 3:**
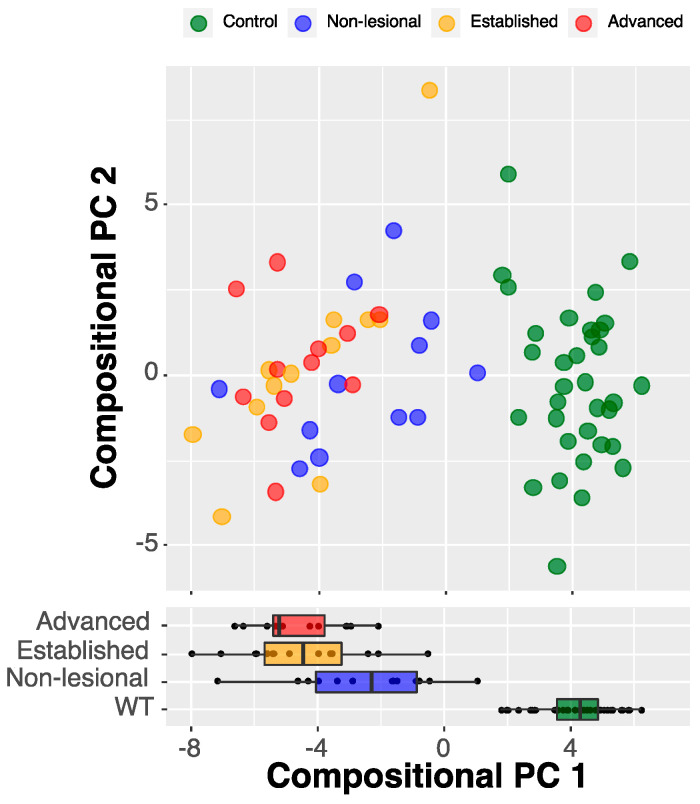
Lipid ions delineate disease stage groups. CPC analysis of all lipid ions plotted vs. disease progression. The model was able to delineate the controls and the three experimental groups of *cpdm* mice (η^2^ = 0.86, *p*-value < 0.001).

To study epidermal lipid changes associated with disease progression, a multiclass case was considered instead of a binary case. The *cpdm* mice were further divided into subclasses defined by the disease stage as non-lesional, established, and advanced. For general visualization of the data, we first computed CPC values using as input only the lipid data pre-selected in the previous binary step with the ENET filtering. The plot was prepared using the disease stage markings in a CPC space demonstrated that such a simple model was able to partially delineate the controls (independently of their age) and the levels of the *cpdm* genotype (Figure 3).

#### 2.2.2. Compressed-Feature Selection for Disease Progression

**Figure 4:**
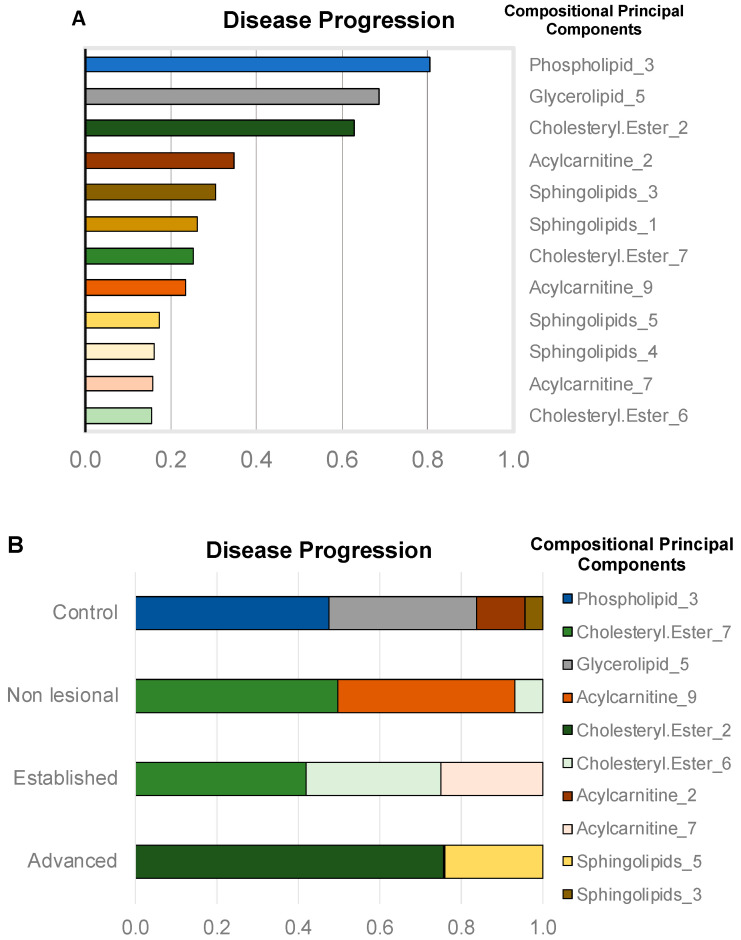
Importance of compressed categories for disease progression classification. (**A**) univariate linear model—selected lipid categories CPC based on their effect size (n^2^). (**B**) contribution of the top 10 CPC to the classification of disease progression categories by the multivariate elastic net model with an accuracy of 81%.

The disease-progression analysis, performed in a univariate setting, pointed to phospholipids CPC 3, glycerolipids CPC 5, and cholesteryl esters CPC 2 as the most informative compressed features (Figure 4A). The top features associated with disease progression produced η^2^ ranging from 0.63 to 0.8. It is important to note that the features predicting disease progression were the same as those that separated WT from the broad *cpdm* group containing animals in all disease stages. The feature selection and ranking task performed by the ENET again identified phospholipid CPC 3, cholesteryl esters CPC 2, and glycerolipids CPC 5, as the top features in terms of importance. Interestingly, the highly ranked features were not equally important for all the disease stages (Figure 4B).

The disease progression prediction with an ENET classifier using a multinomial model achieved an overall accuracy of over 0.81, CI_95%_ = (0.71, 0.9). The substantial part of the observed inaccuracy was caused by the high similarity between samples from the adjacent “established” and “advanced” stages of the disease. This effect is also demonstrated by the difference between the unweighted and weighted Cohen’s κ values (0.725 and 0.841, respectively).

#### 2.2.3. Individual Lipid Ions Feature Selection for Disease Progression

**Figure 5:**
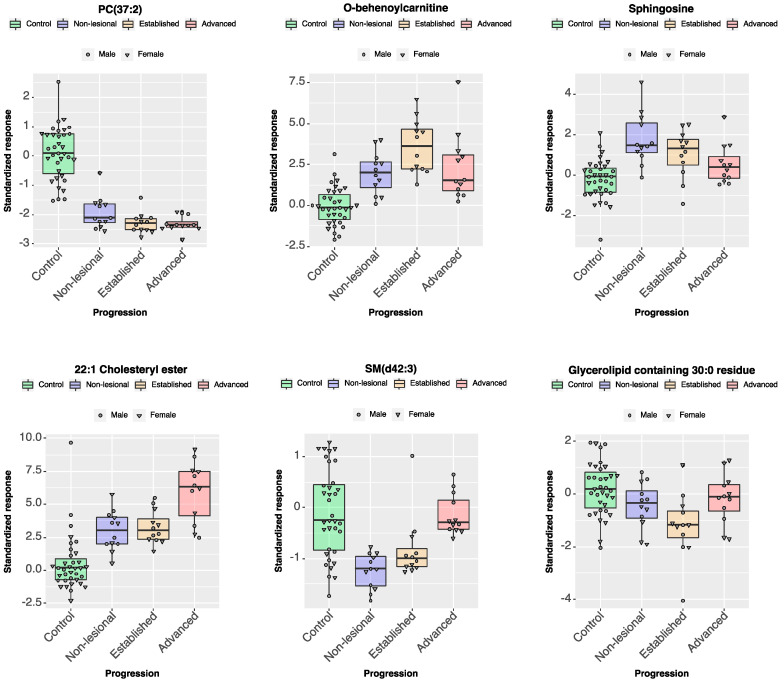
Epidermal lipid ions predictive of disease progression in mice. Representation of six lipids from the epidermis of WT and *cpdm* mice identified as predictive of disease stage in a sex-independent manner. Lipid features emphasize differences between controls and the various stages of the disease.

The univariate feature selection step for disease progression selected ions with η^2^ ranging from 0.22 to 0.73 and phospholipids dominated the very top of the list. The following multivariate analysis, performed by training an ENET, found a more diverse set of ions, some of them distinctly associated with a particular disease stage, but less useful for predicting others. It is an expected characteristic of a multivariate model, which combines all the features and their predictions to form a functional classifier. The ions contributing highly to the prediction of progression are listed in Table 3, and the results are illustrated in Figure 5.

**Figure 6:**
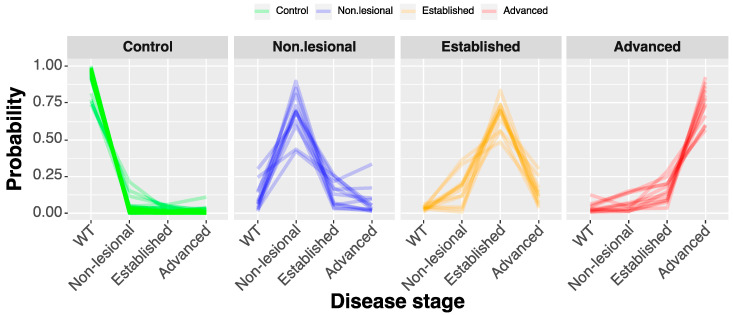
Classification of samples into disease progression groups. Parallel plot illustrating the classification of individual samples by elastic net regression. Each line represents a sample, and the highest point in the line corresponds to the group where the sample would be classified with higher probability.

The ENET classifier trained on the disease progression data was able to classify the 36 *cpdm* and 36 WT samples into groups, including the control and the three disease stages with an overall accuracy of 0.79, CI_95%_ = (0.67, 0.87) when classified using weighted classes and 0.95, CI_95%_ = (0.88, 1) if the synthetic minority sampling technique (SMOTE) algorithm was used for correcting the class imbalance (Figure 6). The training used the variations transformed features corresponding to the presence of phosphatidylcholines, cholesteryl esters, acylcarnitines, and a glycerolipid-containing triacontanoic acid fatty acyl residue.

## 3. Discussion

Lipids comprise a highly diverse group of molecules that play an essential role in the biology of the skin, and the relative proportions of different lipids are associated with the normal physiological functions of this organ [40,41,42]. In this study, we analyzed the relation between the lipids detected by MRM-profiling (lipidomic profile) and the observed genotypes using two machine learning feature-selection approaches. First, we computed a set of compressed features using compositional principal components to represent each of the lipid categories analyzed. These features easily separated male and female samples indicating a strong influence of sex on the epidermal lipid composition in mice. The first CPC summarizing variance in all the lipids was associated with clustering by sex, rather than by genotype. This result shows that the biochemical variability related to sex was dispersed among many lipids creating an effect more substantial than the one associated with the genotype. This result is in agreement with studies of skin-surface lipid clusters in humans where samples from males and females were distinguishable, but no significant difference between atopic or healthy subjects was observed [23,43]. However, it is necessary to note that the large variance visualized by CPC 1 (Figure 1) does not unequivocally demonstrate the importance of the differences between lipid composition in males and females, as it may also emerge from the fact that the sexual dichotomy has a high signal-to-noise ratio in the lipidomics data. 

The multivariate analysis performed by the ENET demonstrated that classification to the male and female group was influenced mostly by sphingolipids (specifically, the compressed feature sets CPC 4 and CPC 5). The biological function of sphingolipids is determined by their composition, particularly the type of sphingoid base and the number of carbons and hydroxyl groups on the acyl chains, and their synthesis is affected by gonadal hormones in mice [44]. Several studies have shown alterations in ceramides, a sphingolipid, in the epidermis of AD patients [17,45,46,47]. However, conflicting results have been reported for changes in ceramides in non-lesional skin, probably because not all ceramide species are altered at the same stage of the disease and/or by the same mechanisms in males and females [23,48]. Our results show that sexual dimorphism is related strongly to the relative amounts of epidermal lipids in mice, and suggest that sex-related differences in the lipid biology of AD should be further investigated as they may partially explain the contradictory results regarding changes in ceramides in AD patients’ skin [47].

A comparison of lipid categories in the *cpdm* and WT phenotypes by either univariate method or ENET showed differences driven by phospholipids and glycerolipids. The alteration in the lipid composition of the epidermis is a hallmark of AD associated with impaired barrier function of the skin [41,49], but whether these changes are primary or caused by the inflammatory process remains elusive [50,51]. Lipidomic and transcriptomic analysis of atopic patients have shown a global alteration of fatty acids caused by the interrelationship of type 2 cytokines and lipid elongase enzymes [52]. Our analysis demonstrates that the presence of phospholipids and glycerolipids in the epidermis was altered before any clinical signs of disease in the skin of the *cpdm* mice. Phospholipids and glycerolipids are essential for cellular and subcellular membrane dynamics and share common metabolic intermediates [53]. Phospholipids can also be secreted in the lamellar bodies of the epidermis along with the enzymes that use them as a substrate for ceramide synthesis [54]. Alterations in their concentrations may affect skin barrier function, cell metabolism, and inflammatory cell signaling, as they can carry esterified fatty acids that generate lipid mediators of inflammation by undergoing fatty acyl remodeling [55]. Likewise, lipids resulting from an aberrant lipid metabolism may be incorporated into membranes as phospholipids are in constant flux [55,56]. In agreement with our results, changes in phospholipids were reported to be present in the serum of atopic patients compared with controls [57]. In another mouse model of AD, NC/Nga mice, phospholipids were decreased in plasma, and oral supplementation of plasmalogens increased the phospholipid concentration in the skin of the mice and improved the skin condition [58]. In human skin, one study reported an increase of phospholipid content in AD patients compared to healthy subjects [24], while others showed a global change in phospholipids with an increase in the presence of shorter acylated fatty acids [52]. In addition to species differences, the disparities in sample preparation approach (epidermis vs. total skin), variations in analytical methods, and identification of the individual lipid species may account for these inconsistent results. 

Changes in different categories of lipids were associated with stages of the disease as the severity of dermatitis increased. The control epidermis could be discriminated from the *cpdm* samples mostly by phospholipids and glycerolipids. However, other lipid categories were required to separate the disease stages of the *cpdm* mice samples. Classification of stages was influenced by acylcarnitines, cholesteryl esters, and sphingolipids. Acylcarnitines are fundamental for β-oxidation by delivering fatty acids to mitochondria and peroxisomes as an energy source; therefore, their dysregulation could potentially redirect fatty acids towards increased biosynthesis of phospholipids and other lipids, rather than being used as a source of energy [59,60]. Acylcarnitines were found dysregulated along with the phospholipid content in the serum of atopic patients [57]. Acylcarnitines also play a role in inflammatory processes, and their accumulation has been linked to lipotoxicity that results in apoptosis [61]. Cholesteryl esters, like acylcarnitines, are carriers of fatty acids and serve to store cholesterol in lipid droplets for its transport [62,63]. Free cholesterol is an essential constituent of the epidermis [19]; however, cholesteryl esters are less polar than free cholesterol conferring the skin with enhanced hydrophobicity for the barrier function [64]. Increased levels of free cholesterol in the skin of atopic patients [51], along with reduced levels of cholesteryl esters associated with high-density lipoproteins [65] suggest alterations in systemic cholesterol homeostasis, as reported in cardiovascular diseases [66]. The data showing an influence of sphingolipids on the classification model reproduces our previous results in which ceramides were identified as necessary for the classification of samples from *cpdm* mice with advanced dermatitis [31]. Changes in ceramide content are correlated with impaired barrier function and increased transepidermal water loss [51,67]. They are also associated with the disease severity, resulting in considerable changes in lesional compared to non-lesional skin [68]. Interestingly, sphingosine was an exception as its concentration was increased more in non-lesional than in lesional skin.

This study demonstrated an association between the presence of particular lipids in the epidermis and the occurrence of chronic proliferative dermatitis in mice. The link was displayed by a data-driven selection of MRM-extracted lipid features, training of a classifier, and identifying the key features contributing to the high classification accuracy. Although the presented procedure relies entirely on a statistical model, we hypothesize that the selected features will contribute to mechanistic insights if studied further. This argument is built upon the notion that the lipids providing high classification accuracy must be involved in the processes causing the phenotypic changes. Given that the lipid composition fingerprint differed not only between visibly lesional *cpdm* skin specimens and controls but was also altered in asymptomatic samples, the identified lipids could be considered candidates for predictive disease biomarkers. The current study involved an exploratory screening design that compares the lipid profiles of the groups using similar amounts of samples. The data analysis was performed employing compositional (relative) representation. Our study’s scope did not include the validation of informative lipids by LC-MS/MS with the addition of internal standards; therefore, the isotopic and isobaric overlap may have occurred, and other lipids than the ones labeled with tentative attributions may have contributed to the reported predictive features. However, our previous work utilizing the relative amounts of ceramides demonstrated a concurrence between the relative values and the results obtained using quantitative LC-MS/MS [31]. Our analysis showed that not a single lipid (or lipid category) is modified sufficiently to be the sole differentiating factor. The accurate separation between healthy and diseased animals required an entire vector of lipid features, including phospholipids, acylcarnitines, cholesteryl esters, and sphingolipids. This result points to a multifaceted and multivariate nature of AD-associated lipid alterations in the skin.

The reliance on a classification tool to extract the most predictive lipids also defines the limitation of the presented approach. The task becomes particularly challenging when facing a severe class imbalance, as in the case of disease progression [69]. It is evident that sample availability determines the training performance, which in turn affects the robustness of the feature selection. We are aware of this limitation and hope that continuing research will allow for larger sample sizes, and correspondingly more confident analysis.

## 4. Materials and Methods

### 4.1. Animals

72 male and female C57BL/KaLawRij-*Sharpin^cpdm^/Sharpin^cpdm^* RijSunJ (*cpdm*) mice and WT littermates were obtained from the Jackson Laboratory and housed at 2 to 4 animals per box with food (Envigo) and water ad libitum. Room temperature was maintained at 20 ± 2 °C and relative humidity at 50 ± 15% with a 12/12-h light/dark cycle. Then, 18 WT males and 18 WT females and their *cpdm* littermates were divided into three groups of six males and six females with different ages and disease stages. The disease progression corresponded to non-lesional (5 weeks of age), established (7 weeks), and advanced (10 weeks) stages. Mice from the non-lesional group had no clinical signs of dermatitis on the dorsal or abdominal skin. The mice in the established group displayed erythema, moderate scaling, and mild alopecia of the dorsal and ventral skin. At 10 weeks, dermatitis covered most of the body with significant hair loss, erythema, thickening, and scaling. Mice were euthanized at 5, 7, or 10 weeks of age by CO_2_ asphyxiation and cervical dislocation. The animal experiments and procedures were conducted in accordance with the Guide for the Care and Use of Laboratory Animals of the National Institutes of Health. The protocol was approved by the Purdue University Animal Care and Use Committee (PACUC protocol 111001019).

### 4.2. Epidermis Isolation and Lipid Extraction

Sample collection and lipid extraction were performed as previously described [31]. Briefly, a 1 by 2 cm slice of dorsal skin was collected, and after incubation with Thermolysin (from *Geobacillus stearothermophilus*, Sigma-Aldrich, St. Louis, MO, USA) dissolved in HEPES buffer, the epidermis was peeled off and stored at −80 °C until extraction. Tissue was weighed and homogenized in 250 µL of ultra-pure water using Precellys24 tissue homogenizer (Bertin Technologies, Rockville, MD, USA). The homogenate was submitted to a Bligh and Dyer [70] liquid–liquid extraction, and the organic phase was collected and dried in a concentrator. Samples were resuspended in 40 µL of 3:1 (*v*/*v*) acetonitrile (ACN)/chloroform, then diluted 50× with ACN/methanol/ammonium acetate 300 mM at 3:6.65:0.35 volume ratio for mass spectrometry analysis.

### 4.3. MRM-Profiling Method Development and Sample Screening

A composite sample of each group from the testing set was created by pooling aliquots of 5 µL from each specimen in the group. The composite samples were analyzed using a previously described methodology of MRM-profiling discovery experiments [31]. Briefly, neutral loss (NL) and precursor ion (Prec) scans were used to profile phospholipids, acylcarnitines (AC), sulfatides, cholesteryl esters, ceramides, glycerolipids with diverse fatty acid acyl residues, triacylglycerides, and free fatty acids in positive and negative ion modes [31,71,72,73,74,75]. Using a micro-autosampler (G1367A), 8 µL of the sample was directly delivered into a QQQ6410 triple quadrupole mass spectrometer (Agilent Technologies, San Jose, CA, USA) equipped with an ESI ion source. A cap pump (G1376A) was used to flow acetonitrile plus 0.1% formic acid at a rate of 5 µL/min. The source capillary and multiplier voltages were 3500 V and 300 V, respectively. The collision energy voltage was 2 V for the negative ion mode methods. In positive ion mode, the collision energies varied according to the lipid classes. For ceramides, phosphatidylethanolamines (PE), and lipids with arachidonate acyl residue and oleate acyl residue, the collision energy was set at 22 V, for phosphatidylcholines and sphingomyelins (SM) at 20 V, for phosphatidylserines (PS) and phosphatidylinositols (PI) at 16 V, for CE at 17 V and for acylcarnitines the collision energy was set at 30 V. The fragmentation voltage of all the methods was 100 V. In total, 80 different discovery scans were performed, producing 1030 informative lipid ions. The parent and the fragment were collected and organized as transitions in 6 different methods of 2 min each (Table S2). The individual samples were flow-injected 6 times to cover all the monitored lipid ions. The raw data files are deposited in the public proteomics repository MassIVE (http://massive.ucsd.edu) using the identifier: MSV000083884. The tentative identification of lipid ions was performed through MS/MS experiments and by using reference databases, such as the Lipid Maps database (http://www.lipidmaps.org/) and METLIN (https://metlin.scripps.edu). Validation of the method by liquid chromatography-mass spectrometry has been previously reported along with the linearity and dynamic range of over four orders of magnitude from 1 to 10,000 ppm [31].

### 4.4. Data Analysis

Using MSConvert (http://proteowizard.sourceforge.net), the files were converted into the mzML open-source format, and an in-house script was used to obtain the ion intensities of each *m*/*z* monitored. The relative amounts of each *m*/*z* were used for data analysis.

The visualization and subsequent selection of lipid categories /groups and individual lipid ions associated with the *cpdm* genotype were performed following normalization of the signals to 1 using the total ion count and subsequent transformation using the isometric log-ratio function or centered log-ratio function. For visualization of all the overall characteristics of the data, the pre-processed input was compressed using singular value decomposition. The resultant first two compositional principal components were employed to illustrate the tendency of the samples to separate themselves in the reduced dimensionality space into clusters according to the sex, the genotype, and the disease severity [76].

#### 4.4.1. Selection of Predictive Lipid Categories

To identify the categories of lipids associated with the sex or genotype, the pre-processing and compression were followed by a two-tier selection including a univariate step, and a multivariate step driven by ENET regression.

In the beginning, the measured lipid ions were annotated and assigned to one of the following categories: (1) acylcarnitine, (2) acylcarnitine or glycerolipids, (3) cholesteryl esters, (4) DAG, (5) glycerolipids, (6) phospholipids, (7) phospholipids or cholesteryl esters, (8) phospholipids or glycerolipids, (9) sphingolipids, and (10) sphingolipids or glycerolipids. The overlap in categories reflects the uncertainty of the attribution due to the use of only one MRM related to a lipid candidate. Each of the sub-dataset was compressed using SVD to create compressed features sets. The number of retained columns (and by extension, the number of principal components used to represent the categories) was selected to retain 95% of the variance in each category. Therefore, the number of reduced composite features per class varied from 4 to 21. The resultant composite features for every lipid class were named “CPC”, followed by the component number. For instance, “sphingolipids CPC 4” denotes the fourth principal component of the log-ratio transformed sphingolipids-class data.

It is important to emphasize that the SVD of the compositional data was not utilized here to enable a PCA-driven feature selection, but rather to produce a highly compressed input for the separate feature selection step. In other words, we are not claiming a direct association between the lipids that happen to display the most variation and the lipids (or lipid classes) that are most likely to be predictive and biologically significant.

The described data reduction process resulted in the creation of 57 compressed lipid-class features describing each of the samples. Subsequently, 57 linear models linking the computed features with sex, and another 57 models linking the features with the genotype (*cpdm* vs. control) were created. Finally, the third set of 57 linear models was computed to relate the features with the disease progression of *cpdm* mice (using the class assignment of control < non-lesional < established < advanced disease status). Benjamini–Hochberg *p*-value adjustment [77] was used to correct for false discovery. The features associated with genotype models having *p*-value < 0.05 (and η^2^ effect sizes ranging from 0.73 to 0.1) were picked for the further feature selection step. For the sex-dependent changes in lipids, we also picked features with *p*-value < 0.05 (and η^2^ effect sizes from 0.22 to 0.12).

#### 4.4.2. Feature Selection of Predictive Individual Lipid Ions

In a similar procedure, in order to recover the most predictive individual lipid ions (rather than lipid categories), we first created 1,030 linear models linking the log-ratio transformed relative amounts of every transition to genotype and sex. We pre-selected the features that might be associated with disease progression (either in sex-dependent or sex-independent manner) by selecting *p*-value < 0.01 for the criteria that were included and *p*-value > 0.05 for the factors that were ruled out. The lipids present in linear models connecting significantly with disease progression after Benjamini–Hochberg *p*-value adjustment (*p* < 0.01), but not being significant for sex (*p* > 0.05) were selected as sex-independent predictive lipids. About 50 ions were selected as possibly predictive and represented η^2^ effect sizes ranging from 0.724 to 0.29.

#### 4.4.3. Predictive Elastic Net Regression

The final but critical step of these feature selection procedures involved the use of ENET regression [78]. ENET was employed either as a binary (for sex and *cpdm* vs. WT separation) or a multiclass classifier. This regression approach includes LASSO *L*_1_ and ridge *L*_2_ penalty terms leading to a predictive model operating in a reduced dimensionality of the data produced by the MRM-profiling:

(1) $$\hat{\beta }=\underset{\beta }{\mathrm{argmin}}({\left\|y-X\beta \right\|}^{2}+\lambda ((1-\alpha ){\left\|\beta \right\|}^{2}/2+\alpha {\left\|\beta \right\|}_{1}))$$

In the ENET formula above, the input matrix **X** consists of all the pre-selected measured lipid ions (or pre-selected composite lipid categories), the output vector **y** describes the stages of the disease, and α, λ ≥ 0 are tuning parameters. The penalties included in the mathematical model are in the ‖β‖_1_ term which generates a sparse model by shrinking some regression coefficients to zero and, in the ‖β‖^2^ term which removes the limitation on the number of selected variables but encourages grouping effect, allowing similar features to be selected together. The individual lipid ions or the composite lipid categories with the larger absolute value of β are considered to be more predictive. The ENET simplifies to ridge regression when α = 1 and to the LASSO regression when α = 0.

The ENET regression was trained using the leave-one-out approach. Due to significant data imbalance, we used class weights (imposing a lesser penalty for errors in the majority class) or the SMOTE approach during training [79]. The resultant classifier allowed us to rank the lipid ions in terms of importance (ability to influence ENET prediction) using the absolute value of the non-zero coefficients.

To visualize the changes in the selected features, a central log-ratio transformation followed by standardization to the female WT subgroup was performed. Therefore, the y-axis in the figures shows the difference in relative lipid abundance as the number of standard deviations away from the WT-female group. The multiclass ENET prediction was illustrated using a parallel plot.

The statistical analyses were performed using R-language for statistical computing.

## 5. Conclusions

In this study, we paired an exploratory high-throughput lipidomics technique with rigorous machine learning analysis to rapidly screen for potential biomarkers in a mouse model of dermatitis. The measurements were performed using flow injection to the ion source of a triple quadrupole mass spectrometer, providing highly sensitive, but low-resolution mass data. The exploratory approach relied on product ions and neutral losses expected to be specific to the lipid classes, but not individual lipids; therefore, the detected lipids are assigned only tentative attributions. The approach revealed sexual dimorphism in the epidermal lipid profile, which was distributed throughout the lipid categories and identified sphingolipids as the best predictors for sex classification. Furthermore, epidermal lipid analysis allowed accurate classification of samples, not only by the genotype of the mice, cpdm vs. WT, but by the stages of disease progression. A panel of lipids comprised of phospholipids, acylcarnitines, sphingolipids, and cholesteryl esters was necessary to achieve successful classification into the different disease stages, showing that a single lipid or lipid category was not altered sufficiently to be the sole classifier. These results highlight the need to consider sex-related differences in the pathobiology of AD and the importance of building lipid panels that include lipids from different categories when investigating predictive biomarkers for AD.

## Figures and Tables

**Table 1:** List of top 10 lipid ions ranked by the effect size of the univariate models linking genotype with the lipidomic profile.

Category	Tentative Attributions	MRM	Genotype (η^2^)	Sex (η^2^)
Phospholipid	PC(37:2), PC(O-38:2), PC(P-38:1)	800.6→184.1	0.76	0.001
Phospholipid	SM(d41:1) *	801.6→184.1	0.72	0.002
Sphingolipid	Cer[AS](d18:1/24:0)2OH	666.4→264.3	0.63	0.025
Phospholipid	PC(38:2), PC(P-39:1)	814.6→184.1	0.63	0.018
Phospholipid	SM(d36:0) *	733.6→184.1	0.61	0.010
Phospholipid	SM(d42:1) *	815.6→184.1	0.60	0.001
Phospholipid	PC(38:1), PC(P-38:1), PC(O-38:2) *	816.6→184.1	0.60	0.022
Phospholipid	PC (32:1), PC(O-33:1), PC(P-33:0)	732.1→184.1	0.59	0.008
Phospholipid	PC(40:8), PCo(40:1)	830.1→184.1	0.57	0.014

* Subject of possible isotopic interferences.

**Table 2:** List of top lipid ions ranked by importance score for prediction of genotype using the elastic net model.

Category	Tentative Attributions	MRM	Importance Score
Phospholipid	PC(37:2), PC(O-38:2), PC(P-38:1)	800.6→184.1	100.00
Glycerolipid	Glycerolipid containing 22:5 residue	627.1→280	50.58
Sphingolipid	Cer[AS](d18:1/24:0)2OH	666.4→264.3	48.40
Phospholipid	PC(38:1), PC(P-38:1), PC(O-38:2)	816.6→184.1	32.26
Phospholipid	SM(d41:1) *	801.6→184.1	29.57
Phospholipid	PC(38:2), PC(P-39:1)	814.6→184.1	19.86
Glycerolipid	Glycerolipid containing 18:2 residue	895.1→598	3.35
Phospholipid	SM(d37:0)	745.6→184.1	0.61

* Subject of possible isotopic interferences.

**Table 3:** List of top 10 lipid ions ranked by importance score for prediction of disease progression in the elastic net model.

Category	Tentative Attributions	MRM	Importance Scores				
			Control	Non-Lesional	Established	Advanced	Overall
Phospholipid	PC(37:2), PC(O-38:2), PC(P-38:1)	800.6→184.1	100.00	0.00	0.00	0.00	100.00
Acylcarnitine	O-behenoylcarnitine	484.4→85.1	0.00	0.00	77.26	0.00	77.26
Sphingolipid	Sphingosine	300.2→282.2	0.00	71.50	0.00	0.00	71.50
Phospholipid	PC(31:0), PC(O-31:1), PC(P-31:0)	720.4→184.1	0.00	0.00	0.00	66.55	66.55
Cholesteryl Ester	22:1 Cholesteryl ester	725.4→369.1	0.00	0.00	0.00	60.59	60.59
Sphingolipid	Cer(d27:2)	438.2→ 266.2	0.00	0.00	44.09	0.00	44.09
Glycerolipid	Glycerolipids containing 30:0 residue	624.1→155.1	0.00	0.00	42.86	0.00	42.86
Phospholipid	SM(d42:3)	811.6→184.1	0.00	30.66	0.00	7.66	38.32
Acylcarnitine	Non attributed	837→85.1	0.00	36.96	0.00	0.00	36.96
Phospholipid	PC (36:0), PCp(38:6)	790.4→184.1	0.00	26.66	0.00	0.00	26.66

## Supplementary Materials

The following are available online at https://www.mdpi.com/2218-1989/10/7/299/s1, Table S1: Transitions for compositional principal component (CPC) score plot, Table S2: Compiled method for individual analysis of samples.
